# Rare Run-Over Clinical Case. Evisceration of the Intestines through the Wrist

**DOI:** 10.15388/Amed.2025.32.2.9

**Published:** 2025-12-30

**Authors:** Jekaterina Strelčenko, Diana Vasiljevaitė, Sigitas Chmieliauskas, Sigitas Laima, Jurgita Stasiūnienė, Paulius Petreikis

**Affiliations:** 1 Faculty of Medicine, Vilnius University, Vilnius, Lithuania; 2 Department of Pathology, Forensic Medicine, Institute of Biomedical Sciences of the Faculty of Medicine of Vilnius University, Vilnius, Lithuania; 3 Department of Pathology, Forensic Medicine, Institute of Biomedical Sciences of the Faculty of Medicine of Vilnius University, Vilnius, Lithuania; 4 Department of Pathology, Forensic Medicine, Institute of Biomedical Sciences of the Faculty of Medicine of Vilnius University, Vilnius, Lithuania; 5 Department of Pathology, Forensic Medicine, Institute of Biomedical Sciences of the Faculty of Medicine of Vilnius University, Vilnius, Lithuania; 6 Department of Anatomy, Histology and Anthropology, Institute of Biomedical Sciences, Faculty of Medicine, Vilnius University, Vilnius, Lithuania

**Keywords:** run-over, visceral evisceration, multiple traumas, blunt trauma, autopsy, forensic pathology, pervažiavimas, vidaus organų evisceracija, dauginiai sužalojimai, buka trauma, autopsija, teismo medicina

## Abstract

**Background:**

Deaths resulting from car-pedestrian collisions are frequently encountered in forensic practice. Such accidents often cause extensive, multi-system injuries, many of which are concealed beneath minimally damaged soft tissues and may not be immediately visible externally. Common external injuries include extensive skin abrasions, subcutaneous hematomas, and lacerations. Internally, these incidents typically result in multiple bone fractures and severe organ ruptures. A less frequent but noteworthy injury is the evisceration of abdominal organs through ruptures in the diaphragm, rectum, or abdominal wall. This report presents a unique case of small intestine evisceration through damaged skin on the wrist.

**Materials and methods:**

The literature search was conducted in the *PubMed* and *Google Scholar* databases using keywords. The analysis focused on scientific literature that was published in last 10 years but also referred to older scientific papers with strong arguments. To the best of our knowledge, there are no reported cases of evisceration of abdominal contents through the damaged skin of the lower arm (wrist). A clinical case examination from the Lithuanian State Forensic Medicine Service data was performed.

**Case presentation:**

A 70-year-old woman was injured in the run-over accident by a truck. The victim died at the scene. External examination revealed multiple injuries, including significant soft tissue deformities in the left arm. During the internal examination of the corpse, multiple fractures with ruptures of internal organs were found. Only isolated fragments of intestinal loops were present in the abdominal cavity. Evisceration of the small intestines through the left upper arm and forearm was found.

**Conclusions:**

Run-over accidents involving heavy vehicles are a common cause of fatal injuries. These incidents often result in extensive lacerations, multiple comminated fractures, and severe damage to internal organs. In rare cases, evisceration of internal organs can occur through various anatomical sites. This case demonstrates that evisceration can occur not only through common locations such as the diaphragm, abdominal wall, rectum, or vagina but also through atypical sites, such as the skin of the wrist. In such cases, it is particularly important for a forensic pathologist to assess the damage of clothing and to evaluate the macromorphological appearance of the injuries to determine the mechanism of injury.

## Abstract (it)

**Įvadas:**

Mirties atvejų, kai automobilis partrenkia ir sužaloja pėsčiąjį, teismo medicinos gydytojo praktikoje nėra reta. Tačiau pervažiavus judančios transporto priemonės ratais nukentėjusįjį padaroma dauginių masyvių sužalojimų, daugelio jų išoriškai ir nematoma. Tipiniai išoriniai sužalojimai – masyvūs odos nubrozdinimai, poodinės kraujosruvos, plėštinės žaizdos. Dažniausiai nustatomi vidiniai sužalojimai yra dauginiai skeveldriniai kaulų lūžiai, masyvūs vidaus organų plyšimai. Retesnis sužalojimas pervažiavimo atvejais yra pilvo organų evisceracija per diafragmos plyšimą, tiesiąją žarną arba pilvo sienelę. Mūsų darbe pateikiamas retas plonųjų žarnų evisceracijos per riešo odą atvejis.

**Medžiaga ir metodai:**

**:** Mokslinės literatūros apžvalga atlikta *Pubmed, Google Scholar* duomenų bazėse. Daugiausiai analizuota 10 metų senumo mokslinė literatūra, tačiau remtasi ir senesniais, turinčiais svarių argumentų mokslo darbais. Mūsų žiniomis, pilvo turinio evisceracijos per pažeistą rankos (riešo) odą atvejų nėra aprašyta mokslinėje literatūroje. Išanalizuotas Valstybinės teismo medicinos tarnybos nuasmenintas klinikinis atvejis.

**Atvejo pristatymas:**

70 metų moteris buvo partrenkta ir pervažiuota sunkvežimio. Sužalota nukentėjusioji mirė įvykio vietoje. Išorinio tyrimo metu, be kitų sužalojimų, nustatyta kairėje rankoje daugybė minkštųjų audinių deformacijų. Lavono vidinio tyrimo metu buvo nustatyta dauginių skeveldrinių lūžių ir vidaus organų plyšimų. Pilvo ertmėje buvo matoma tik pavienių žarnų kilpų fragmentų. Nustatyta plonųjų žarnų evisceracija per kairį žastą ir dilbį.

**Išvados:**

Nelaimingas atsitikimas, kai nukentėjęs asmuo partrenkiamas ir pervažiuojamas sunkiasvoriu automobiliu, dažniausiai baigiasi mirtimi. Pervažiavimas transporto ratais gali lemti masyvių plėštinių žaizdų susiformavimą, dauginius skeveldrinius kaulų lūžius su vidaus organų plyšimais ir retais atvejais jų evisceraciją per įvairias kūno sritis. Mūsų atvejis rodo, kad evisceracija gali įvykti ne tik per minėtas dažniausiai anatomines lokalizacijas, tokias kaip diafragma, pilvo sienelė, tiesioji žarna ar makštis, bet ir per neįprastas kūno sritis, pavyzdžiui, riešo odą. Tokiais atvejais teismo medicinos gydytojui yra ypač svarbu įvertinti drabužių pažeidimų bei kūno sužalojimų makromorfologinį vaizdą, kad būtų galima nustatyti sužalojimų mechanizmą.

## Introduction

Deaths caused by car-pedestrian collisions are a routine part of a forensic pathologist’s daily routine, especially in areas where urbanisation is prevalent. Run-over accident is an accident in which a person is involved in the impact of a moving vehicle or a rolling object. This mechanism of injury often results in multiple trauma, which is often hidden under a slightly damaged soft tissue cover (skin, subcutaneous tissue). Typical external signs include extensive bruising, skin abrasions and burns. For internal injuries, the most common mechanism is blunt crush injuries. Rarely, run-over trauma can lead to evisceration of organs through different areas of the body, such as the abdominal wall, vagina, anus, or diaphragm. We present a unique case of evisceration of the small intestines, through damaged skin in the wrist.

## Methods

The literature search was conducted in the *PubMed* database and the online *Google Scholar* search engine by using keywords relevant to the subject, including ‘multiple trauma’, ‘blunt trauma’, ‘visceral evisceration’, ‘run-over’, ‘autopsy’. The analysis focused on scientific literature that was published in last 10 years but also referred to older literature or scientific papers with strong arguments. To the best of our knowledge, there are no reported cases of evisceration of abdominal contents through the damaged skin of the lower arm (wrist). 16 pertinent articles were selected. Furthermore, all run-over accidents recorded by Vilnius Department of the Lithuanian State Forensic Medicine Service over the past 10 years were analysed. Among these cases, only one was found to involve similar injuries. Data provided by the Lithuanian State Forensic Medicine Service were used in the analysis, which included a detailed examination of the clinical case. Detailed information on case was provided by law enforcement agencies, encompassing vital details such as the incident location, the time of death, and the presumed cause of death. A complete autopsy was conducted to thoroughly investigate the circumstances of the death. Following the forensic dissection, blood and urine samples were systematically collected for alcohol and drug testing. Headspace gas chromatography techniques were employed to detect the presence of alcohol, providing crucial insights into the toxicological profile of each case.

## Case presentation

**Fig. 1:**
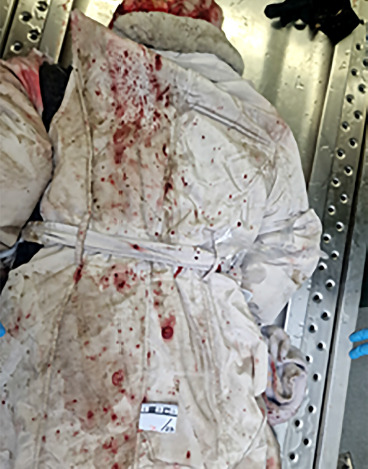
Repeated dirt smears repeating a possible tyre tread pattern.

**Fig. 2:**
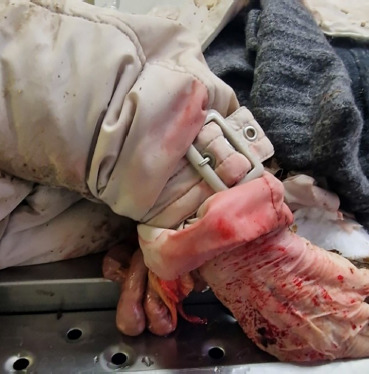
Small intestine loops herniation visible through the jacket sleeve.

**Fig. 3:**
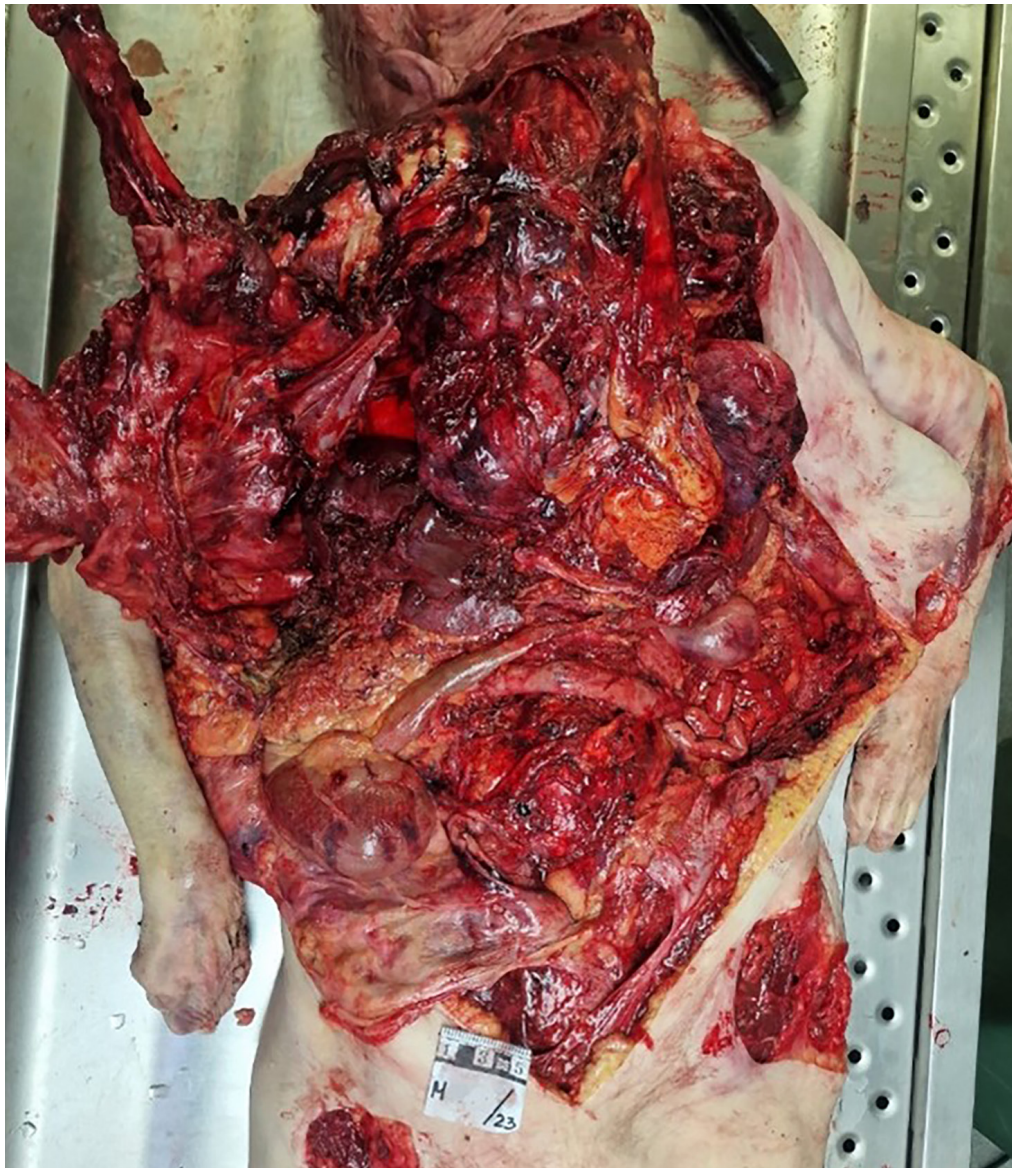
A small amount of remaining bowel visible in the abdominal cavity.

**Fig. 4:**
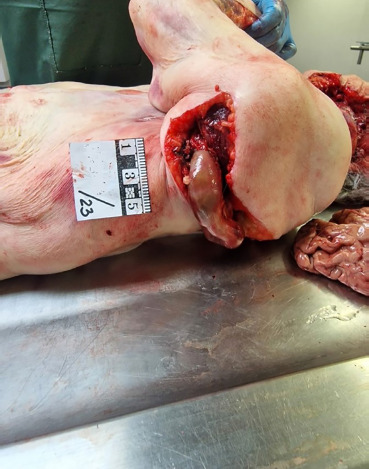
Partial prolapse of the intestines through the laceration on the back and upper third of the arm (external view).

**Fig. 5:**
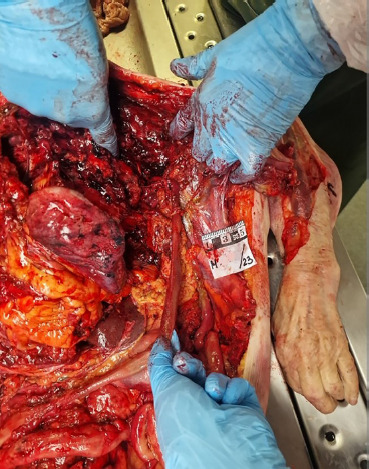
Partial prolapse of the intestines through the laceration on the back and upper third of the arm (internal view).

**Fig. 6:**
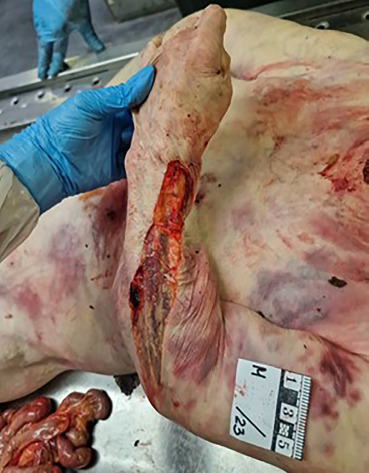
The intestine extends in a pocket formed between the fascia and subcutaneous fat of the dorsal side of the left upper arm and forearm. Several loops of intestine prolapse through a laceration in the left wrist (external view).

**Fig. 7:**
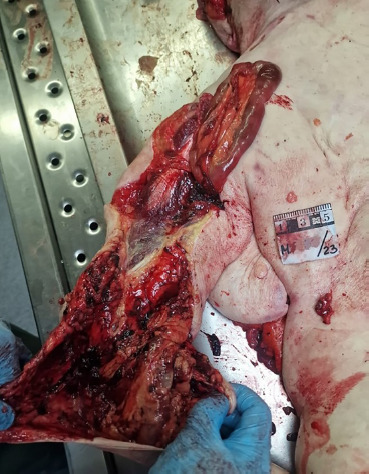
The intestine extends in a pocket formed between the fascia and subcutaneous fat of the dorsal side of the left upper arm and forearm. Several loops of intestine prolapse through a laceration in the left wrist (internal view).

A 70-year-old woman was run over by a truck. Ambulance arrived and found the woman unconscious under the wheels. The victim died at the scene. Dirt and soil stains on the front surface of the outer clothing (jacket and trousers) and traces of dried blood on the back surfaces were present. On the back surface of the jacket, in a diagonal direction (from the lower right edge, upwards towards the upper left shoulder), repeated dirt smears were observed at intervals of 0.8 cm, at 3 to 3.5 cm in width, repeating a possible tyre tread pattern (Fig. 1). The left sleeve of the jacket was prolapsed with intestinal loops (Fig. 2). The left arm was deformed, with multiple soft raised bumps, resembling the relief of intestines. Internal examination of the corpse revealed internal organs (heart, lungs, large blood vessels, liver, spleen, pancreas, kidneys and adrenal glands, pelvic organs) with multiple lacerations. Only fragments of intestinal loops were observed in the abdominal cavity (Fig. 3). Part of the small intestine prolapsed through a laceration wound on the left side of the pelvis. The other part of the small and large intestines extended upwards along the left wall of the abdominal cavity, through the ruptured diaphragm, towards the inner surfaces of the ribs of the thoracic ribs on the left, and extended to the left upper arm, partially prolapsing through the laceration on the dorsal surface of the upper one-third of the upper arm and back (Fig. 4, Fig. 5). The intestine then extended into a narrow pocket formed between the fascia and subcutaneous fat of the dorsal surface of the left upper arm and forearm. (Fig. 6, Fig. 7). Several loops of intestine prolapsed through the laceration on the left wrist. The injuries found were caused by a hard blunt object, whereas mainly compression, torsion, and tension mechanisms were present.

**Table 1:** Mechanisms of injury

Autopsy findings	
External examination	Dirt and soil stains on the front surface of the outer clothing repeating a tire tread pattern; multiple lineal parallel abrasions and haematomas on the skin; laceration of the skin and soft tissue on the back and upper third of the arm
Internal examination	Multiple fractures of the cranial vault, skull base, and facial bones; fractures of both clavicles; displaced multiple rib fractures; spinal fractures between the bodies of the 3^rd^ and 4^th^ thoracic vertebrae, between the 4^th^ and 5^th^ thoracic vertebrae, between the 10^th^ and 11^th^ thoracic vertebrae; fractures of the spinous processes of the 1^st^, 2^nd^, and 5^th^–11^th^ thoracic vertebrae; fractures of the spinous processes of the 4^th^ and 5^th^ lumbar vertebrae; fractures of both scapulae; fracture of the left humerus; multiple fractures of the pelvic bones and sacrum; fracture of the right femur; bilateral fractures of the lower leg bones (tibia and fibula); multiple fractures of the right ankle bones; extended pericardium tearing; multiple lacerations of the heart with complete artery rupture in its origin; multiple tears in both lungs with bilateral haemothorax; diaphragmatic rupture; multiple tears of the liver; multiple tears of the spleen; multiple tears of the pancreas; multiple tears of the kidneys and adrenal glands; multiple tears in the intestinal loops with dislocation in the thoracic cavity; displacement of the intestine into a pocket formed between the fascia and subcutaneous fat of the left upper arm and forearm.
**Vehicle’s data**	During the run over, the truck’s weight was 29,500 kg The rear axle had wheels of the type 315/70 R22 Each tyre had a width of 315 mm
**Mechanisms of injury**	The rear wheel ran along the subject’s abdomen and chest. The vehicle’s wheels were moving at a low speed from bottom to top in relation to the victim, which led to pushing out the internal organs.

## Discussion

Deaths caused by car-pedestrian collisions are a regular part of a forensic pathologist’s daily routine, especially in areas where urbanisation is prevalent [1]. Although most pedestrian-vehicle collisions involve a vehicle striking a pedestrian, there are also cases where pedestrians are at risk of being run over by a vehicle [2]. A run-over is an accident in which a person is subjected to the load of a moving vehicle or a rolling object. Often, this mechanism of injury results in multiple injuries, which are often hidden under a slightly damaged soft tissue covering (skin, subcutaneous tissue). Typical external signs include extensive contusions, abrasions, skin lacerations, compression injuries, and burn [3]. For internal injuries, blunt trauma is the most common mechanism of injury. Blunt trauma is defined as an injury to the body caused by the mechanical force exerted by the impact of a moving blunt object or by the movement of the body against a hard surface, both of which transfer sufficient kinetic energy to cause an injury (mainly compression, torsion, and/or traction) [4]. The mechanism of injury in a motor vehicle run-over includes:

- impact by the wheels of the motor vehicle;
- friction of the wheels on the clothing, body;
- friction of the body (clothing) on the road;
- compression of the body between the wheel and the pavement.

The forces of acceleration and deceleration cause stretching and tearing between fixed and mobile structures, resulting in damage to organs, connective tissue, blood vessels, and nerves [5].

The detection and analysis of any tyre tread marks on clothing and/or the body is an essential element in the investigation of accidents where the victim may have been run over. A clear tread mark provides valuable information in several respects:

- the position of the victim before the accident;
- the areas of the body affected;
- the path of the wheel in relation to the body axes;
- the characteristics of the tread pattern (e.g., the number, position and width of grooves) [5, 6].

As mentioned above, when a victim is run over, the mechanism of blunt trauma is usually visible. Rarely, blunt trauma may result in organ evisceration [7].

Evisceration of abdominal organs through the abdominal wall is quite common in adults after penetrating trauma. Evisceration due to blunt trauma is unusual. The most common sites for organ evisceration are the abdominal wall, vagina, anus, and diaphragm. Transabdominal (abdominal wall) hernia associated with blunt trauma is very rare, with an estimated incidence of 0.2–1% in the main series described [8]. Abdominal organ evisceration is even less common, occurring in approximately 1 in 40 000 cases. Abdominal evisceration is an extreme form of abdominal wall hernia and, like other hernias, it usually occurs in anatomically weak areas such as the lateral, lower abdomen and inguinal regions [9].

The literature also describes several cases of small bowel prolapse through the rectum or ileum after being run over by car wheels. The rectum is a fixed organ located below the peritoneum, while the ileum is an intraperitoneal, flexible organ. Trauma causes a sudden increase in abdominal pressure and a difference in shear forces, which leads to a rupture of the intraperitoneal rectum or colon at its weakest point. Usually, there is a longitudinal tear along the antimesenteric margin. The small intestine passes through this tear and collapses through the anus [10, 11].

In addition to damage to the organs of the lower abdomen and the abdominal wall, a sudden increase in intra-abdominal pressure can cause injury to the diaphragm (hernia and rupture), which can lead to organs sliding through the diaphragm into the upper body [12]. Diaphragmatic injuries are relatively rare, occurring in less than 1% of all traumatic injuries [13]. Approximately 80% of all hernias and ruptures occur on the left side, probably due to the relative weakness of the left diaphragm and the protective effect of the liver on the right diaphragm [14]. Ruptures occur at sites of potential weakness, usually at the musculotendinous junction. Various organs can pass from the abdominal cavity to the thorax through diaphragmatic ruptures, most commonly the pancreas, stomach, large intestine, and spleen [15].

It is of importance to note that the severity and consequences of most injuries are determined not only by the mechanism, but also by the state of the body’s tissues. The scientific literature shows that ageing is associated with increased fascial stiffness and reduced elasticity, as well as a decrease in the skeletal muscle mass, strength and regenerative potential. The interaction between muscles and fascia weakens with ageing. This may partly explain the mechanism of the injuries caused by the trauma described in our case. The reduced elasticity of the fascia and the weakened connective tissue-muscle bond may lead to easier breaks and tears in the fascia, which may result in internal organs (e.g., intestines) being trapped in the resulting gaps [16].

## Conclusions

Being run over by a heavy car is usually a fatal accident. The human body is severely injured due to the high force: extensive lacerations, fractures of various bones, multiple lacerations of internal organs, and, in rare cases, evisceration of internal organs through various body regions. Our case shows that evisceration can occur not only through the common places such as the abdominal wall, rectum or vagina, but also through the more unusual ones such as the wrist. In such cases, it is particularly important for a forensic pathologist to assess the damage and stains of clothing and to evaluate the macromorphological appearance of the injuries in order to determine the mechanism of injury.
